# Socio-economic and spatial correlates of subclinical iodine deficiency among pregnant women age 15–49 years in Tanzania

**DOI:** 10.1186/s40795-017-0163-1

**Published:** 2017-06-05

**Authors:** Abdalla H. Mtumwa, Julius Edward Ntwenya, Edwin Paul, Megan Huang, Said Vuai

**Affiliations:** 1 Department of Statistics, The University of Dodoma, P.O. Box 338, Dodoma, Tanzania; 2 Department of Public Health, The University of Dodoma, P.O. Box 395, Dodoma, Tanzania; 3 Department of Chemistry, The University of Dodoma, P.O. Box 338, Dodoma, Tanzania; 4 Department of Public Health, University of Dodoma, P.O. BOX 259, Dodoma, Tanzania

**Keywords:** Iodine deficiency, Pregnancy, Iodized salt

## Abstract

**Background:**

Iodine deficiency is a widespread global health problem that affects about 2 billion people each year. Pregnant women are particularly vulnerable to iodine deficiency due to increased iodine requirement leading to death, miscarriage, and stillbirth. Iodine deficiency also has significant negative effects on newborns including impaired cognitive development, impaired learning capabilities, and stunting. This study looks at the association between subclinical iodine deficiency and demographic factors including age, wealth index, education, family size, geographical zone, number of children, fish consumption, pregnancy trimester and household salt in pregnant women aged 15–49 years in Tanzania.

**Methods:**

The 2010 Tanzania Demographic and Health Survey (TDHS) data was re analysed. Subclinical iodine deficiency is classified as a urinary iodine concentration (UIC) of <150 *μ*g/L.

**Results:**

Results showed that the prevalence of iodine deficiency (54%) was unacceptably high among pregnant women. The results of multiple logistic regression model found that number of children, wealth index, household salt, and geographical zone were significantly associated with iodine deficiency in these women.

**Conclusion:**

These results indicate a need to implement interventions to increase iodine intake that targets pregnant women with the specific demographic characteristics.

## Background

In the past decade there has been a great focus on increasing iodine intake, but iodine deficiency remains a major health problem around the world. In the year 2011, it was estimated that about 2 billion people in the world had inadequate iodine intake [1]. Iodine deficiency particularly impacts young children and pregnant women especially in developing countries. About 38 million young children suffer from iodine deficiency in developing countries every year [2–4] and national surveys suggest that about 20–65% of pregnant women are iodine deficient in developing countries [5–8].

Iodine deficiency has severe effects on pregnant women and newborns [9, 10]. These include death to women during childbirth, miscarriage, stillbirth, congenital abnormalities and intellectual dysfunction [11, 12]. Iodine deficiency may also lead to brain damage and poor growth in newborns leading to lifelong negative effects on cognitive development and learning abilities [2, 11, 12].

A 1999 report by WHO and UNICEF indicated that 44 out of 46 sub-Saharan African countries, including Tanzania, had high prevalence of iodine deficiency [13]. A study done in Ethiopia in 2014 has shown that overall median UIC in pregnant women was 58.1*μ*g/L and 82.8% of the women had subclinical iodine deficiency [14]. Another study conducted in Congo in 2013 revealed that the overall median UIC in pregnant women was 138 (inter quartile range: 105–172) mg/l, indicating iodine deficiency [15]. In Tanzania it was estimated that 41% of the population lived in areas exposed to iodine deficiency were iodine deficient [16]. Despite of iodine intake improvement made s in Tanzania following the implementation of universal salt iodization (USI) in 1985 when all salt for human consumption was fortified with iodine [17]; many still lack access to adequately iodized salt, and prevalence of iodine deficiency remains high especially among pregnant women [18]. Studies on iodine deficiency have been conducted in Tanzania, but most of the studies focused on the general population, young children and women aged 15–49 without giving special consideration to only pregnant women [16, 19–23].

A number of studies have been conducted in Western countries such as France [24] and Kosovo [25] as well as in Mali [26] and Bangladesh [27] to establish the association between women’s iodine nutritional status and several socio-economic characteristics. These studies found a significant association between women’s iodine nutritional status and demographic characteristics such as wealth, socio-economic status, and education and modern contraceptive use though results were mixed on the relationship between age and iodine nutritional status. In Kosovo, the relationship between age and iodine nutritional status was found to be not significant, whereas in studies conducted in France, Mali, and Bangladesh, age and iodine nutritional status were found to be significantly associated.

Thus recent studies on the association between women’s socio-economic characteristics with their iodine nutritional status in pregnant women aged between 15 and 49 years have had wide coverage but are limited in Tanzania [28]. Therefore, there is a need for further investigation into factors that influence iodine deficiency. The present study aimed to determine the association between socio-demographic factors and subclinical iodine deficiency among pregnant women aged between 15 and 49 years. This study will enrich the availability of information and contribute to developing appropriate interventions for reducing iodine deficiency among pregnant women in Tanzania.

## Methods

### Data description

The study used data from the nationally representative 2010 Tanzania Demographic and Health Survey (TDHS). The survey maintained all the protocols prescribed by the World Health Organization [29]. The survey was designed to provide data to monitor the population and health situation in Tanzania as a follow up to the previous four TDHS conducted during 1991–2005. The survey utilized a multistage cluster sample based on the 2002 Tanzania Census and was designed to produce separate estimates for key indicators for each of the eight geographic zones of the country: Western (Tabora, Shinyanga, Kigoma), Northern (Kilimanjaro, Tanga, Arusha, Manyara), Central (Dodoma, Singida), Southern Highlands (Mbeya, Iringa, Rukwa), Lake (Kagera, Mwanza, Mara), Eastern (Dar es Salaam, Pwani, Morogoro), Southern (Lindi, Mtwara, Ruvuma), and Zanzibar (Unguja North, Unguja South, Town West, Pemba North, Pemba South). Data collection began on 19 December 2009 and was completed on 23 May 2010.

The survey obtained detailed information on household characteristics, fertility levels and preferences, awareness and use of family planning methods, childhood and adult mortality, maternal and child health, breastfeeding practices, antenatal care, childhood immunization and diseases, nutritional status of young children and women, malaria prevention and treatment, women’s status, female circumcision, sexual activity, knowledge and behavior regarding HIV/AIDS, and prevalence of domestic violence. The survey collected information of a weighted sample of 11,224 women aged 15–49 years. The sample was weighted by the weighting factor provided in the survey data. Of the women, 10,260 were currently not pregnant and 17 had missing information regarding urinary iodine concentration (UIC). Thus a total of 10,277 women aged 15–49 years were excluded from the analyses leading to a sample size of 947 women. The details of the survey are well-described in the 2010 TDHS report [30].

### Outcome variable

The unit of analysis of this study was ‘subclinical iodine deficiency’ which was measured from UIC. WHO recommends urinary iodine concentrations of <150, 150–249, 250–499, and ≥500 *μ*g/L to indicate insufficient, adequate, more than adequate, and excessive levels of iodine intake respectively in the pregnant women population [31]. In this study, UIC <150 *μ*g/L defined subclinical iodine deficiency and ≥150 *μ*g/L defined absence of subclinical iodine deficiency. The group with <150 *μ*g/L was further categorized into <20 *μ*g/L (severe iodine deficiency), 20–49 *μ*g/L (moderate iodine deficiency), and 50–149 *μ*g/L (mild iodine deficiency) [32].

### Explanatory variables

Previous studies revealed that a number of socio-demographic, cultural, political and environmental factors affect women’s iodine nutritional status. Household salt is an important factor in analyzing iodine nutritional status. In this study, household salt was classified as “iodized” and “not iodized”. The current age of the study women was classified into three categories: 15–24, 25–34 and 35–49 years. Since women’s education is an important factor for nutritional status, it was broken down into three categories: no formal education, primary education, and secondary or higher education. One of the background characteristics used in this study was the household economic status namely ‘wealth index’. The wealth index used in this study was developed and tested in a large number of countries to measure inequalities in household income, use of health services, and health outcomes [33]. It is an indicator of the level of wealth that is consistent with expenditure and income measures [34]. The wealth index was constructed from data on household assets, including ownership of durable goods (such as televisions and bicycles) and dwelling characteristics (such as source of drinking water, sanitation facilities, and construction materials). To create the wealth index, each asset was assigned a weight (factor score) generated through principal component analysis, and the resulting asset scores were standardized in relation to a normal distribution with a mean of zero and standard deviation of one [35]. Each household was then assigned a score for each asset, and the scores were summed for each household; individuals were ranked according to the total score of the household in which they resided. The sample was then divided into quintiles from one (poorest) to five (richest). The eight geographic zones of the country – Western, Northern, Central, Southern Highlands, Lake, Eastern, Southern and Zanzibar –were selected as another background variable in this study to assess zonal variation in iodine deficiency status of pregnant women. The family size was grouped as a dichotomous variable namely ‘<5 persons’ and ‘≥5 persons’.

### Statistical methods

To examine the relationship between subclinical iodine deficiency status and background characteristics of the women, UIC was made a binary response. If a woman was iodine deficient, she was coded as ‘1’ and coded ‘0’ otherwise. In doing so, the women with more than adequate, and excessive levels of iodine intake were coded ‘0’. Fixed effect logistic regression analyses were used in this study. Simple logistic regression model was applied to examine the association between background characteristics and subclinical iodine deficiency while a multivariable logistic regression model was applied to assess the net effects of the selected factors on ‘subclinical iodine deficiency’ among pregnant women. The results of the regression analyses are presented by odds ratios (OR) with 95% confidence interval (CI) for easy understanding of the effect of the corresponding factor after controlling for other confounders. Statistical analyses were performed using SAS version 9.4.

### Ethics

Ethics approval was granted to the National Bureau of Statistics (NBS) by the Tanzania’s National Institute for Medical Research, the Zanzibar Medical Research and Ethics Committee, the Institutional Review Board of ICF International, and the US Centers for Disease Control and Prevention. Informed consent was obtained individually from all respondents before the start of the interview. Permission to re-analyze and publish the findings was granted by the NBS.

## Results

### Background profile of the respondents

A total of 947 pregnant women were included in the analysis. The mean age of the respondents was 26.89(SD ± 6.89) years. About 50% of the respondents reported using salt with inadequate iodine content, and 637(67.27%) were living in a families with more than five people. With regard to education, 226(23.92%) had no formal education; the majority, 587(62.12%), had primary education; and 132(13.97%) had completed at least a secondary level of education. In terms of wealth index, about 41% were poor, less than one-third were from middle class families and about 36% were rich. More women, 21.33%, were from the Zanzibar zone and fewest, 6.23% were from the Central zone. Our study found that about 50% of the cooking salt samples were adequately iodized. The characteristics of women participating in this study are detailed in Table 1.

**Table 1:** Characteristics of pregnant women in Tanzania, 2010 TDHS

Variable	Total frequency (%)	Iodine deficiency frequency (%)	No Iodine deficiency frequency (%)	*P* Value*
Age (Years)				0.4500
15–24	399(42.13)	207(51.88)	192(48.12)	
25–34	385(40.65)	213(55.32)	172(44.68)	
35–49	163(17.21)	93(57.06)	70(42.94)	
Total	947(100)			
Education Level				0.0006
No education	226(23.92)	128(56.64)	98(43.36)	
Primary education	587(62.12)	333(56.73)	254(43.27)	
Secondary or higher	132(13.97)	51(38.64)	81(61.36)	
Total	945(100)			
Wealth Index				<0.0001
Poorest	160(16.9)	103(64.38)	57(35.63)	
Poorer	229(24.18)	150(65.50)	79(34.50)	
Middle	215(22.7)	122(56.74)	93(43.26)	
Richer	210(22.18)	107(50.95)	103(49.05)	
Richest	133(14.04)	31(23.31)	102(76.69)	
Total	947(100)			
Iodine content in household salt				<0.0001
Inadequate	457(50.22)	315(68.93)	142(31.07)	
Adequate	453(49.78)	181(39.96)	272(60.04)	
Total	910(100)			
Family size				0.0009
< 5 persons	310(32.73)	144(46.45)	166(53.55)	
≥ 5 persons	637(67.27)	369(57.93)	268(42.07)	
Total	947(100)			
Geographic zone				<0.0001
Eastern	72(7.6)	14(19.44)	58(80.56)	
Western	169(17.85)	117(69.23)	52(30.77)	
Northern	97(10.24)	39(40.21)	58(59.79)	
Central	59(6.23)	34(57.63)	25(42.37)	
Southern Highlands	94(9.93)	50(53.19)	44(46.81)	
Lake	163(17.21)	88(53.99)	75(46.01)	
Southern	91(9.61)	72(79.12)	19(20.88)	
Zanzibar	202(21.33)	99(49.01)	103(50.99)	
Total	947(100)			
Number of children				0.0003
0	209(22.40)	88(42.11)	121(57.89)	
1–4	559(59.91)	323(57.78)	236(42.22)	
5+	165(17.68)	96(58.18)	69(41.82)	
Total	933(100)			
Fish consumption				0.0106
< 1 day/Week	360(38.01)	214(59.44)	146(40.56)	
≥ 1 day/Week	587(61.99)	299(50.94)	288(49.06)	
Total	947(100)			
Pregnancy trimester				0.3605
First	267(28.19)	139(52.06)	128(47.94)	
Second	348(36.75)	199(57.18)	149(42.82)	
Third	332(35.06)	175(52.71)	157(47.29)	
Total				

* *P* values are based on likelihood ratio chi-square test

### Subclinical iodine deficiency among pregnant women

**Fig. 1:**
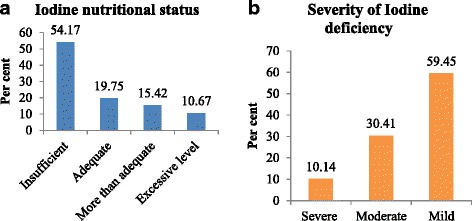
Iodine nutritional status and severity of iodine deficiency among pregnant women aged 15–49 years, panels **a** and **b** respectively

The median UIC was 26 *μ*g/L (interquartile range (IQR) = 31–21). When women’s iodine nutritional status was categorized by UIC, about 54% had subclinical iodine deficiency. Of the total respondents, 187 (19.75%), 146 (15.42%), and 101(10.67%) had adequate, more than adequate, and excessive iodine intakes, respectively (Fig. 1, panel a). Among 513 women with subclinical iodine deficiency, 52 (10.14%), 156 (30.41%), and 305 (59.45%) had UIC of <20 *μ*g/L (severe iodine deficiency), 20–49 *μ*g/L (moderate iodine deficiency), and 50–149 *μ*g/L (mild iodine deficiency), respectively (Fig. 1, panel b).

**Fig. 2:**
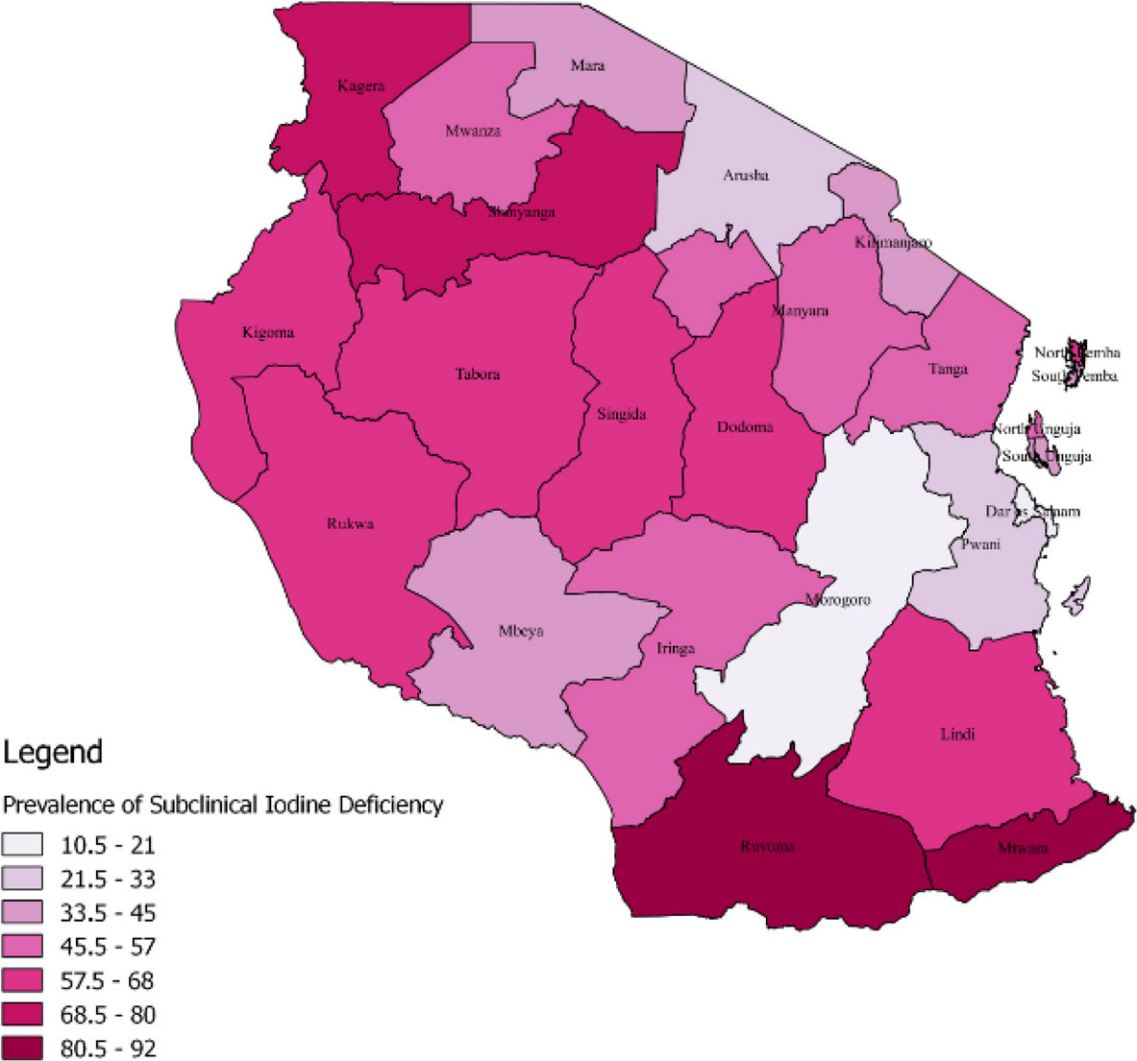
Subclinical iodine deficiency prevalence categories by region of residence among pregnant women of reproductive age based on the 2010 TDHS survey

Among women with no education and with primary education, about 57% were reported to have iodine deficiency, while only 38.64% were iodine deficient among those with secondary or higher education. The likelihood of iodine deficiency was highest among poorer (65.50%) and poorest (64.38%) women, while it was found to be least among the richest (23.31%) women. Almost 70% of women who were using non-iodized salt were reported to have iodine deficiency, while this proportion among women who used iodized salt was 39.96%. The risk of subclinical iodine deficiency was higher among women with a family size of five or more (57.93%) than among those with a family size below five (46.45%). Spatial locations showed wide variation in iodine nutritional status among pregnant women. The prevalence of iodine deficiency was highest in women of the Southern zone (79.12%), followed by women of Western (69.23%), Central (57.63%), Lake (53.99%), Southern Highlands (53.19%), Zanzibar (49.01%), Northern (40.21%) Eastern (19.44%) zones respectively (Table 1). Southern regions of Tanzania had highest prevalence of iodine deficiency among pregnant women of reproductive age. Other regions with the highest prevalence included Kagera and Shinyanga both in the Mainland Tanzania. The prevalence of subclinical iodine deficiency ranged between 33 and 57% for most regions. Regions with minimum subclinical iodine deficiency were Dar es Salaam and Morogoro, with prevalence ranging from 10 to 20% (Fig. 2).

### Correlates of subclinical iodine deficiency among pregnant women

The results of the bivariate analysis presented in Table 2 showed that, the risk of subclinical iodine deficiency was significantly associated with women education level (*p* = 0.0006), family wealth index (*p* < 0.0001), salt iodine content (*p* < 0.0001), family size (*p* < 0.0001), geographic zone (*p* < 0.0001), number of children (*p* < 0.0001), fish consumption (*p* = 0.0029), and pregnancy trimester (*p* = 0.0108).

**Table 2:** Correlates associated with subclinical iodine deficiency in logistic regression model among pregnant women in Tanzania,2010 TDHS

Variable	Crude OR (95%CI)	*P*-Value	Adjusted OR(95%CI)	*P*-Value
Age (Years)		0.1680*		0.2012*
15–24	Reference			
25–34	1.24(0.94–1.63)	0.1335	1.32(0.89–1.95)	0.1671
35–49	1.38(0.92–2.06)	0.1157	0.90(0.48–1.69)	0.7385
Education Level		0.0006*		0.9605*
No education	Reference			
Primary education	0.74(0.54–1.00)	0.0516	0.99(0.69–1.44)	0.9824
Secondary or higher	0.31(0.17–0.56)	0.0001	0.89(0.40–2.02)	0.7862
Wealth Index		<0.0001*		<0.0001*
Poorest	9.48(5.53–16.23)	<0.0001	4.25(2.26–7.99)	<0.0001
Poorer	10.22(6.10–17.12)	<0.0001	5.55(3.02–10.20)	<0.0001
Middle	6.31(3.78–10.51)	<0.0001	3.17(1.77–5.69)	0.0001
Richer	4.68(2.76–7.96)	<0.0001	3.63(2.00–6.58)	<0.0001
Richest	Reference			
Iodine content in household salt		<0.0001*		<0.0001*
Inadequate	Reference			
Adequate	0.28(0.21–0.37)	<0.0001	0.38(0.28–0.53)	<0.0001
Family size		<0.0001*		0.0701*
< 5 persons	Reference			
≥ 5 persons	1.95(1.49–2.54)	<0.0001	1.38(0.97–1.94)	0.0701
Geographic Zone		<0.0001*		<0.0001*
Lake	Reference			
Western	1.99(1.34–2.96)	0.0007	1.73(1.07–2.77)	0.0246
Northern	0.54(0.34–0.85)	0.0075	0.57(0.32–0.99)	0.0463
Central	1.19(0.69–2.05)	0.5248	0.73(0.39–1.37)	0.3236
Southern Highlands	0.68(0.44–1.07)	0.0968	0.76(0.45–1.29)	0.3120
Eastern	0.16(0.09–0.29)	<0.0001	0.23(0.12–0.46)	<0.0001
Southern	3.61(1.96–6.67)	<0.0001	2.37(1.17–4.81)	0.0169
Zanzibar	0.79(0.35–1.79)	0.5666	0.84(0.31–2.30)	0.7343
Number of children		<0.0001*		0.0453*
0	Reference			
1–4	2.23(1.63–3.06)	<0.0001	1.73(1.12–2.67)	0.0136
5+	2.78(1.77–4.35)	<0.0001	1.85(0.92–3.73)	0.0854
Fish consumption		0.0029*		0.7020*
< 1 day/Week	Reference			
≥ 1 day/Week	0.67(0.52–0.87)	0.0029	0.93(0.66–1.32)	0.7020
Pregnancy trimester		0.0108*		0.0983*
First	Reference			
Second	1.33(0.97–1.84)	0.0789	1.26(0.86–1.85)	0.2392
Third	0.84(0.61–1.17)	0.3080	0.85(0.57–1.26)	0.4192

*P* Value with * indicates overall *p*- value

All variables discussed in bivariate analysis were included in a multiple logistic regression model to assess the association between each independent variable and iodine nutritional status while adjusting for other important confounding variables. The results of the fitted multiple logistic regression model revealed that, wealth index (*p* < 0.0001), household salt (*p* < 0.0001), geographical zone (*p* < 0.0001) and number of children (*p* = 0.0453) were significantly associated with iodine deficiency among pregnant women. However, the effects of women’s education, family size, fish consumption and pregnancy trimester were no longer significant (Table 2). The risk of subclinical iodine deficiency was significantly higher among women with poorest (AOR = 4.25,95% CI = 2.26–7.99), poorer (AOR = 5.55,95%CI = 3.02–10.20), middle (AOR = 3.17,95%CI = 1.77–5.69) and richer wealth index category (AOR = 3.63,95%CI = 2–6.58) compared to those in richest category. Another important risk factor was iodine content in the household salt, in which the risk of subclinical iodine deficiency was significantly lower among the women used salt with adequate iodine content (AOR = 0.38, 95% CI = 0.28–0.53). With respect to zone, the chance of having subclinical iodine deficiency among pregnant women living in Western (AOR = 1.73, 95% CI = 1.07–2.77) and Southern (AOR = 2.37, 95% CI = 1.17–4.81) zones was significantly higher as compared to women from Lake zone. On the other hand, women in Northern (AOR = 0.57, 95% CI = 0.32–0.99) and Eastern (AOR = 0.23, 95% CI = 0.12–0.46) zones had significantly lower risk of having subclinical iodine deficiency in comparison to women in Lake zone. Though not significant, women from Central (AOR = 0.73, 95% CI = 0.39–1.37), Southern Highlands (AOR = 0.76, 95% CI = 0.45–1.29) and Zanzibar (AOR = 0.84, 95% CL = 0.31–2.30) zones were also less likely to have subclinical iodine deficiency as compared to those of Lake zone. The prevalence of iodine deficiency was positively associated with number of children (*p* = 0.0453), women with 1–4 children (AOR = 1.73,95% CI = 1.12–2.67) were significantly more iodine deficient compared to those with no children. However, the risk of iodine deficiency among women with five and above children was not significant (AOR = 1.85;95% CI = 0.92–3.73).

## Discussion

Our analysis has shown that the prevalence of iodine deficiency among the surveyed pregnant women was unacceptably high. The existence of a large proportion of pregnant women presenting with subclinical iodine deficiency is indicative of inadequate access to properly iodized salt. Though USI policy in Tanzania passed in 1985 required that all salt in the country be iodized, there have been challenges to full implementation. There is a vast and complex network of salt producers in Tanzania each with different distribution schemes and different access to salt iodization technology. Additionally, there has not been consistent monitoring of iodized salt quality, and as a result, people still suffer from iodine deficiency due to consumption of salt that is insufficiently iodized and inability to access properly iodized salt as well as inability to afford iodized salt and lack of education about iodized salt [36]. Inadequate dietary iodine intake as a result of these factors is particularly dangerous in pregnant women as it increases the risk for unwanted pregnancy outcomes. Children born to iodine deficient mothers are more likely to suffer from hypothyroidism, impaired neurological development, and, in severe cases, cretinism [37].

Recent population studies show that women across the globe are at increased risk of developing iodine deficiency especially during the pregnancy period when iodine requirements are increased at least 50% [38, 39]. Though global estimates are not available, national level studies have been used to estimate the prevalence of iodine deficiency in pregnant women. A study in Kolkata, India found that 37% of pregnant women had insufficient iodine status [38]. Similarly, a higher prevalence of iodine deficiency among pregnant women was reported in Belgium, a mildly-iodine deficient country, where one in six pregnant women were iodine deficient indicating that the pregnancy period makes women especially vulnerable to insufficient iodine intake [7].

In this study, the pregnant women aged 25–34 years had higher odds of presenting with subclinical iodine deficiency. The 25–34 age group represents the most active reproductive age group and the age group during which the most women are pregnant, a time when iodine requirements are significantly greater, or lactating, when much maternal iodine is passed from mother to infant during breastfeeding. The demands of pregnancy and lactation may make the 25–34 age group particularly vulnerable to iodine deficiency. A study in Bangladesh showed that pregnant and lactating women had a higher prevalence of iodine deficiency (37 and 33% respectively) compared to their non-pregnant counterparts (3%) [40]. Similarly, our study has shown that women having one to four children were at more risk to iodine deficiency compared to the ones who have not given any delivery. Thus if left unaddressed, iodine deficiency in this age group may have serious health implications to both the mother and the newly born child [7].

Women belonging to the poorer and poorest wealth index categories had higher odds of presenting subclinical iodine deficiency. In addition, wealth index was associated with both consumption of meat and fish. The consumption of meat and fish in most cases is associated with improved income thus indicates that the poor did not have adequate access to iodine rich foods such as fish which are also considered expensive. Contrary to our expectation, pregnant women who belonged to the poorest wealth category had lower odds of having iodine deficiency compared to the poorer wealth category. The observed difference among poorest and poorer pregnant women suggests a need for in-depth analysis to understand the driving factors that contribute to the observed difference between the two wealth categories.

Consumption of adequately iodized salt in the household was beneficial at reducing the risk of developing subclinical iodine deficiency. The existence of few pregnant women who consumed adequately iodized salt presenting subclinical iodine deficiency highlights the importance of consuming iodized salt in the household and calls for more efforts to 1) ensure that salt quality is standardized to contain the recommended levels of iodine and 2) ensure that households have equal access to properly iodized salt. Finally, a geographical disparity in the prevalence of iodine deficiency was observed among pregnant women. In the Southern zone, the odds of iodine deficiency were significantly higher compared to the Lake zone which may result from the large number of small-scale salt producers in this region that are not closely regulated and produce salt with highly variable iodine content. In a survey of small-scale salt producers, 93% did not have adequate iodine content in their salt [41]. Furthermore, in an attempt to standardize salt content, some salt producers, both small- and large-scale, were provided with salt iodation machines, but investigators found that most salt factories had stopped using them due to high operating costs, inability to repair machines when broken down, and in effective technical support for the machines. Instead, these factories used manual hand spray pumps to iodize the salt yielding highly variable, and oftentimes less than recommended, iodine content in the salt [41]. The Eastern zone, which is also classified as being relatively food rich and economically better off, had the lowest odds of having a pregnant woman presenting with subclinical iodine deficiency. This region may also suffer the consequences of inadequately iodized salt from small-scale salt producers though the presence of large- and medium-scale salt producers may counteract this fact. Additionally, the Eastern zone’s proximity to the coast may allow for greater access to and consumption of fish and sea vegetables that are rich in iodine. However, the relationship between proximity to the coast and iodine deficiency requires further investigation as the Southern zone also has much coastline but has a significantly higher odds of iodine deficiency. The Northern zone had a lower odds of iodine deficiency than the Lake zone. This may be because of concentrated areas of tourism in this zone (i.e. Mount Kilimanjaro, Serengeti, Ngorongoro Crater, Lake Manyara, and Tarangire National Park) and tourism’s impact on health and development. For example, tourism brings employment opportunities and thus increased income to the local people which permeates the public sector, businesses and private households. Likewise, tourism may also bring competition and lower the price of household commodities such as salt [42]. Thus the various consequences of tourism could contribute to reduced iodine deficiency in the local population though the health effects of tourism are numerous and complex and require further study.

### Study limitations

This study would have benefited more by conducting a trend analysis to depict how iodine deficiency have been varying across years as well as including additional variables some of which are not contained in the demographic and health survey report. In addition, it was worth re analyzing this data set irrespective of the time lapse since its release in 2010.

## Conclusion

The overall prevalence of iodine deficiency among pregnant women in Tanzania was 54%. Among the set of independent variables included in this study, women age, socio-economic status, iodine content in household salt, and residing in certain geographic zones were significantly important predictors of iodine deficiency in the study population. Continued efforts towards full implementation of universal salt iodization in Tanzania may be an effective strategy for tackling iodine deficiency.
